# A mesocosm study of oxygen and trace metal dynamics in sediment microniches of reactive organic material

**DOI:** 10.1038/s41598-017-10179-3

**Published:** 2017-09-12

**Authors:** Niklas J. Lehto, Morten Larsen, Hao Zhang, Ronnie N. Glud, William Davison

**Affiliations:** 1 Department of Soil and Physical Sciences, Lincoln University, Lincoln, 7647 Christchurch, New Zealand; 2 Department of Biology, Nordic Centre for Earth Evolution (NordCEE), University of Southern Denmark, Odense M, Denmark; 3 Scottish Marine Institute, Scottish Association for Marine Science, Oban, Scotland; 4 Department of Ocean and Environmental Sciences, Tokyo University of Marine Science and Technology, 4-5-7 Konan, Minato-ku, Tokyo, 108-8477 Japan; 5 Lancaster Environment Centre, Lancaster University, Lancaster, United Kingdom

## Abstract

Deposition of particulate organic matter (POM) induces diagenetic hot spots at the sediment-water interface (SWI). Here we explore the effects of intensive POM degradation for metal mobilization at the SWI. By using a combined planar optode-DGT (diffusive gradient in thin-films) sensor we obtained simultaneous measurements of dissolved O_2_ and trace metal dynamics around an aggregate of reactive organic matter placed on the SWI of a sediment mesocosm. The aggregate induced a rapid, highly localized, decrease in O_2_ concentration, resulting in an anoxic feature at the SWI. Co-located with this feature, we observed intense Fe and Mn mobilization, removal of Co, Ni and Zn and found evidence for the concurrent release and precipitation of Pb within a small confined volume. We also identified two small microniches in the anoxic sediment below the SWI, defined by elevated trace metal mobilization. Differences between the metal release rates in these two microniches indicate that they were formed by the mineralisation of different types of organic matter buried in the sediment. Our results provide direct empirical evidence for the potential importance of POM-induced reactive microniches when considering the fluxes of metals from and within aquatic sediments, and suggest that other elements’ cycles may also be affected.

## Introduction

Deposition of reactive organic material at the sediment water interface (SWI) as particles formed from various biological processes in the water column is a well-recognized component of aquatic carbon fluxes^1–3^. Following settlement on the SWI, they can bring about rapid rates of organic material mineralisation within discrete volumes of sediment^4^. These localized “microniches” can make a significant contribution to organic material turnover in surface sediments, and promote a high degree of variability in the cycling of terminal electron acceptors (TEA) and associated elements^4–6^. They have received significant interest in the literature^7–9^, with much work focusing initially on sulphidic microniches^8, 10^ and more recently localized sinks of reactive nitrogen^6, 11^. These studies have suggested that microniches can make a significant contribution to elemental cycling in aquatic environments, however direct analysis of these processes has been limited by the availability of suitable analytical methods.

Oxidation of reactive organic matter induces changes in redox conditions and the associated solubilisation of Fe and other trace metals has been studied in a variety of benthic environments^12–14^. Understanding of these processes has been built on increasingly sophisticated experimental and numerical modelling techniques^15–18^. However, the level of insight that can be gained from many experimental investigations is limited by methodology that effectively averages solute concentrations across various volumes of sediment. This means that critical information about microniches may often be overlooked^9^. Interpretation of transient chemical gradients occurring at mm or sub mm spatial scales due to small-scale heterogeneity in sediment processes is greatly facilitated if measurements are made in two dimensions (2D) with an appropriate temporal and spatial resolution. As small-scale changes in solutes usually reflect complex interactions between different elements, combined sensors for multiple analytes have the potential to enhance understanding of coupled processes.

Diffusive gradients in thin-films (DGT) and planar optodes are two techniques that have been employed to provide high-resolution measurements of elemental fluxes and their distributions in sediments. DGT measurements have advanced our understanding of phosphorus, trace metal and sulphide cycling^10, 19–21^, while planar optodes have provided valuable information on spatial and temporal O_2_, pH, pCO_2_ and NH_4_^+^ dynamics^22–26^. Recently the two techniques have been used together in a combined DGT-planar optode sensor to measure dissolved O_2_ and trace element mobilization simultaneously. This has provided insight into how their biogeochemical cycles are interrelated in different environments. Williams, *et al*.^27^ identified the release of Fe, Pb and As associated with O_2_ efflux by rice roots growing in an anoxic soil, while Stahl, *et al*.^28^ showed trace element mobilization linked to the presence of relict organic matter and increasing O_2_ concentrations of burrow structures in an otherwise anoxic heterogeneous sediment.

Previous work where fresh organic matter was deployed at the SWI used numerical modelling to suggest that the resulting microniches may make a significant contribution to redox cycling of nitrate and other TEAs following localized oxygen depletion^6^. We now seek to advance this knowledge with experiments designed to test the effects of reactive organic matter deposition on the biogeochemical cycling of Fe and Mn in an otherwise oxic sediment. We hypothesize that localized oxygen depletion induced by the rapid microbial mineralisation of organic matter would result in the reductive dissolution of reactive Fe and Mn (oxyhydr)oxide minerals, and the concomitant release of other trace elements (*e.g*. Co, Ni, Cu, Zn and Pb). To test this hypothesis, we placed three aggregates of organic matter at evenly spaced intervals on the SWI of a sediment mesocosm and used the combined DGT-planar optode sensor to make high-resolution measurements of real time O_2_ dynamics and mobilization of trace metals simultaneously at these locations.

## Results and Discussion

### O_2_ dynamics across the SWI during the deployment

Optode measurements showed a rapid localized O_2_ depletion at the locations of all three aggregates following their deployment. However, we primarily focus our subsequent investigations on the area around the central aggregate formed from freeze-dried *Spirulina* material (Fig. S1), as action of biota at the surface of the sediment visibly disturbed the two other aggregates of organic matter. This activity did not affect the central aggregate. The central *Spirulina* aggregate slowly disintegrated at its deployment location during the experiment. This was due to the slow colonisation and degradation of the aggregate by micro- and meiofauna that physically fragment and rework the aggregate.

**Figure 1:**
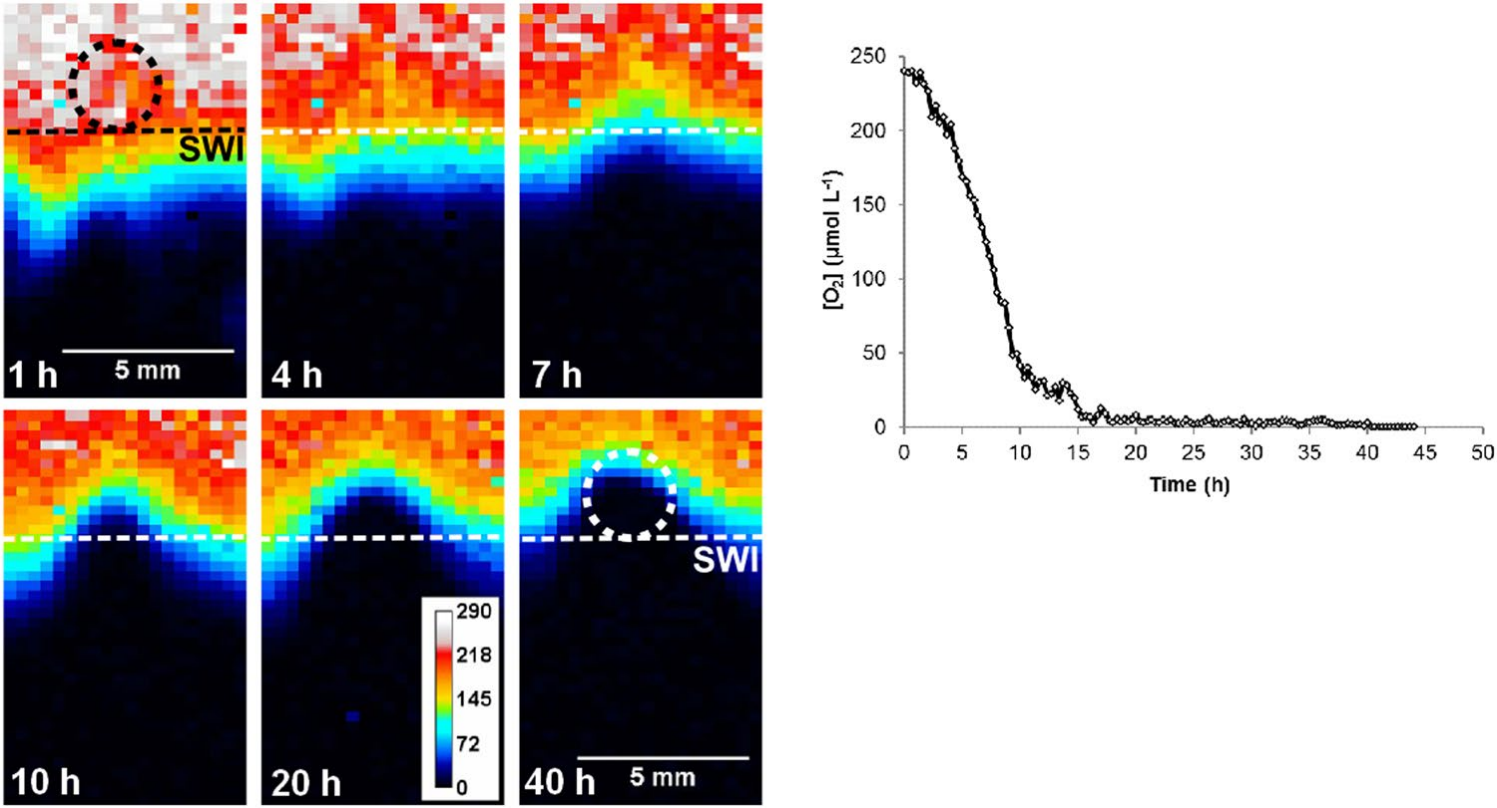
Planar optode measurements of O_2_ concentrations during the deployment (**a**) across the sediment at the location of *I*_x_ and (**b**) at the centre of the aggregate location. The horizontal dashed line shows the sediment-water interface (SWI), the circular dashed line shows the initial position of the aggregate.

The O_2_ concentration decreased rapidly at the centre of the aggregate (Fig. 1). This mimics the dynamic observed in previous investigations of microbial colonisation on parcels of organic matter and is thought to arise from the oxidation of organic matter, resulting in an intense demand for the most energetically favourable TEA (O_2_)^6, 11, 29^. The anoxic isoline, initially beneath the sediment-water interface (SWI), gradually rose upwards towards the centre of the aggregate (Fig. 1a), reaching a distance of 2.2 mm above the original SWI (herein we define the threshold for ‘anoxia’ as the calculated O_2_ method detection limit by the planar optode in this experimental set up, 3.0 µM: see Supporting Information, S.iii.). The anoxic microniche area measured at the probe interface reached a maximum size of ~14 mm^2^ after 44 h, corresponding to approximately half of the aggregate’s cross-sectional area at its centre. Following the emergence of anoxic conditions, the mineralisation of organic matter is thought to be conducted mainly by facultative microorganisms that then employ nitrate as the TEA^6, 14^. Given the low concentration of nitrate in the sediment porewater (1.13 µM, Table S1), and the rapid rate of mineralisation at the location of the aggregate, we believe that the reduction of less energetically favourable TEAs, Mn(IV) and Fe(III), began relatively quickly following anoxia. The location at the centre of the aggregate’s initial position adjacent to the probe had been anoxic for ~32 h when the high-resolution DGT (HR-DGT) was retrieved (Fig. 1b). We estimate that <5% of the organic C in the aggregate was mineralized during the deployment (Supporting Information S1.vi).

The O_2_ concentrations below the SWI decreased rapidly after deployment of the combined sensor and reached a steady state within 100 min. The average background O_2_ penetration depth (*i.e*. depth to anoxic sediment below SWI) and diffusive O_2_ uptake at 43 h were determined from profiles extracted from the O_2_ images (Fig. S1), these were 3.08 ± 0.28 mm (SD, n = 3) and 7.82 ± 0.24 mmol m^−2^ d^−1^ (SD, n = 3): typical for a coastal marine sediment^29^.

### Interpreting DGT flux measurements in the sediment mesocosm

The chelating resin in the HR-DGT probe binds trace metals and thus acts as a planar sink for dissolved trace metals in the sediment and the overlying water. The removal of dissolved metal results in a diffusive flux into the resin from the adjacent environment. The size of that flux is directly related to the dissolved concentration of metal at the interface of the probe and the sediment, as long as the capacity of the chelating resin to bind that metal is not exceeded. When the rate of metal uptake by the HR-DGT exceeds the rate at which the metal can be resupplied from the surrounding sediment, the concentration at the interface, and therefore the flux into the HR-DGT, will progressively decrease. However, when localized processes operating in the sediment close to the HR-DGT interface (*e.g*. reductive dissolution) are rapid and sustained, the mass of solute taken up by the DGT probe can be used to provide an indication of the average metal concentration in the sediment porewater adjacent to the probe during its deployment^30^.

**Figure 2:**
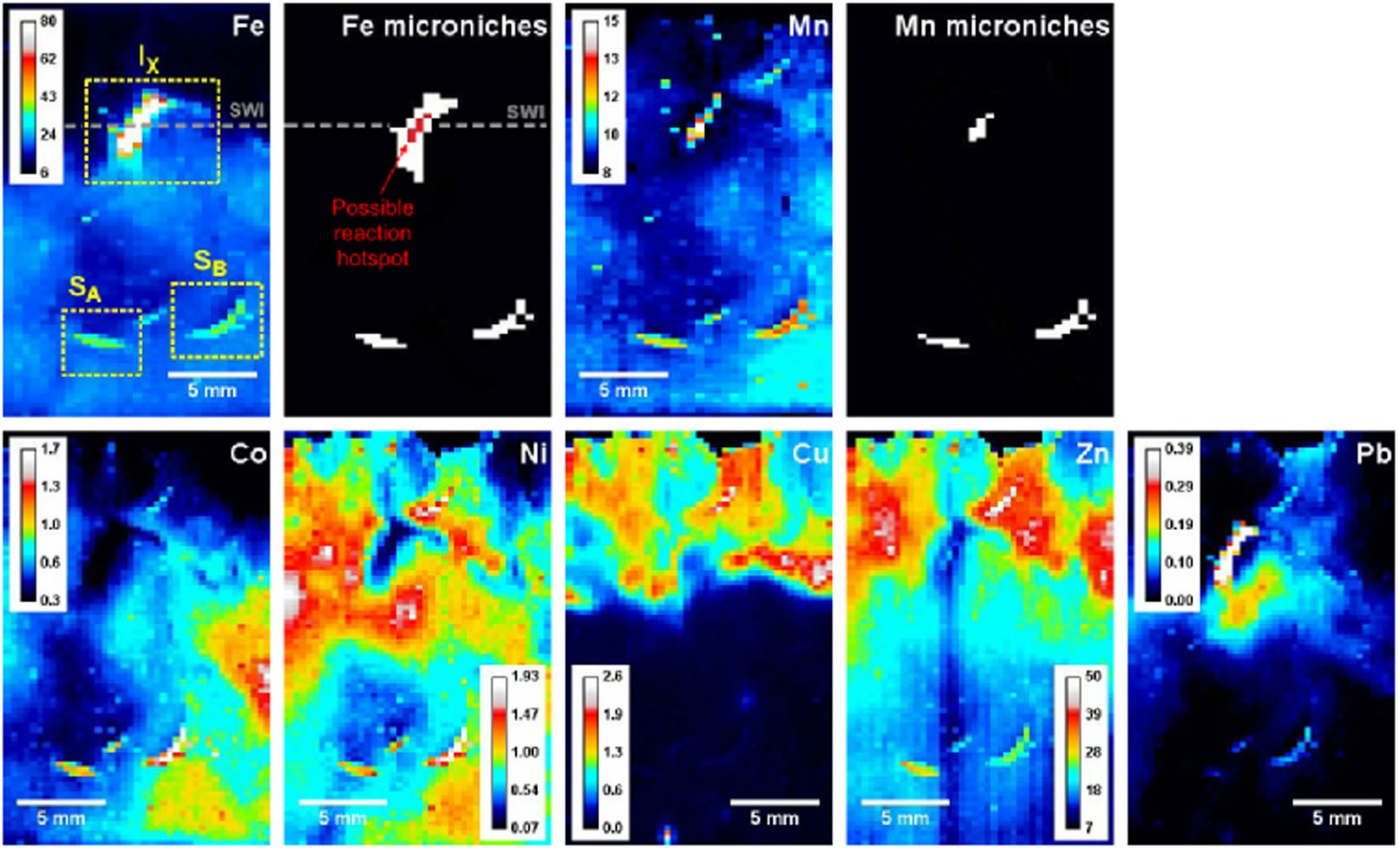
Time-integrated fluxes of Mn, Fe, Co, Ni, Cu, Zn and Pb sediment (fluxes expressed as fmol cm^−2^ s^−1^). The locations of the three microniches, *S*_A_, *S*_B_ and *I*_X_ are shown on the Fe figure. The areas that qualify as Mn and Fe microniches are shown on the monochrome microniche only images; the location of the possible reaction hotspot in *I*_x_ is shown in the Fe microniche image in red. The location of the sediment-water interface (SWI) is shown as the dashed grey line on the Fe images.

The binding of divalent trace metals to the ultra-thin HR-DGT gels used in this work has been shown to be directly related to the metal solution concentration at the device interface when the bound mass is less than 71 nmol cm^−2^ ^31^. The highest total mass of metals measured at any single location in this experiment was 67 nmol cm^−2^. The metal accumulated at any given location in the planar HR-DGT probe is presented here (Fig. 2) for the most part as a time-averaged flux (the mass of metal accumulated by certain area of gel, averaged over the deployment time) to reflect the total supply of metal to the HR-DGT from the sediment and is forthwith expressed as “HR-DGT flux”.

### Distribution of trace metal mobilization below the SWI

To consistently define distinct reference zones, we used areas in the oxic upper 1 mm beneath the SWI apparently unaffected by the aggregates (‘oxic sediment’, total area: 38.6 mm^2^) and the anoxic sediment 6 mm below (‘anoxic sediment’, total area: 517 mm^2^) (Fig. S1). The average background HR-DGT fluxes of Mn, Fe, Co, Ni, Cu, Zn and Pb within oxic and anoxic reference parts of the sediment are presented in Table 1. Background HR-DGT fluxes of Cu, Ni and Zn in the oxic sediment were higher than in the anoxic sediment, while the HR-DGT fluxes of Fe were higher in the anoxic sediment. HR-DGT fluxes of Co, Mn and Pb in the oxic and anoxic sediment were broadly similar (Table 1). The average HR-DGT fluxes of all metals were generally smaller than those observed in previous studies^19, 20, 32, 33^, which is to be expected given the combination of a relatively long deployment time of 44 h and the thinner material diffusive layer used here^31, 34^

**Table 1:** Maximum (Max.) and average fluxes of metals in the bulk oxic and anoxic sediment (the former excludes *I*_x_, the latter includes *S*_A_ and *S*_B_) and the three microniches. The standard deviation of each average is shown in the adjacent brackets. All fluxes are reported as fmol cm^−2^ s^−1^. Superscript letters A, B and C indicate where the average flux of metal across the microniche is significantly different from the other microniches (*p* < 0.05), different letters indicate significant differences between microniches.

		Bulk ‘anoxic’ Sediment (*n* = 3696)	Bulk ‘oxic’ Sediment (*n* = 276)	*I*_X_ (*n Fe* = *53*)	*S*_A_ (*n Fe* = *11*)	*S*_B_ (*n Fe* = *18*)
Fe	Max. Average	49.24 20.02 (±2.96)	13.51 8.63 (±1.33)	384.32 95.40^A^ (±91.71)	37.65 34.87^B^ (±3.21)	38.39 33.53^B^ (±3.28)
Mn	Max. Average	12.87 9.65 (±0.66)	9.76 9.15 (±0.29)	(*n* = 9) 16.31 13.15^A^ (±1.70)	(*n* = 8) 11.77 11.48^B^ (±0.20)	(*n* = 18) 12.87 12.09^B^ (±0.60)
Co	Max. Average	1.66 0.67 (±0.25)	0.72 0.63 (±0.05)	*0.53 0.28* ^A^ (±*0.08*)	*1.10 1.00* ^B^ (±*0.11*)	(*n* = 16) 1.65 1.48^C^ (±0.15)
Ni	Max. Average	2.31 0.81 (±0.25)	1.80 1.40 (±0.17)	*1.29 0.44* ^A^ (±*0.28*)	*1.44 1.07* ^A^ (±*0.20*)	(*n* = 16) 2.00 1.72^B^ (±0.22)
Cu	Max. Average	2.28 0.02 (±0.09)	2.01 1.09 (±0.32)	*1.79 1.15* (±*0.27*)	*N.D*.	*N.D*.
Zn	Max.Average	33.13 15.96 (±3.95)	41.81 34.19 (±3.47)	*22.42 14.95* ^A^ (±*2.07*)	(*n* = 10) 33.13 28.28^B^ (±2.47)	(*n* = 9) 26.53 25.40^C^ (±0.74)
Pb	Max. Average	0.54 0.04 (±0.02)	0.08 0.03 (±0.02)	0.94 0.19^A^ (±0.14)	*0.06 0.02* ^B^ (±*0.19*)	*0.11 0.07* ^B^ (±*0.01*)

Where the flux of a particular metal was not sufficiently elevated above background to qualify it as a ‘microniche’ for that particular metal (*e.g*. Ni for *I*_x_ or *S*_B_), the metal fluxes from the measurement points that define the corresponding Fe microniche were used to enable comparison. These are indicated in italic script, where applicable. Otherwise, the number of measurement points (*n*) is indicated in brackets next to the maximum measured flux for that metal in that microniche. Locations where the mass of metal/unit area is below the detection limit for that metal are designated as “not detected” (N.D.), these were treated as zero concentrations in the calculations of the averages and standard deviations.

.

Depth profiles of O_2_ availability and trace metal release from the sediment showed general patterns broadly similar to those observed in other measurements of benthic trace metal mobilization^19, 35–37^ (Fig. S2). There was little release of Mn, Fe and Co to the HR-DGT near the SWI, but we observed a gradual increase with increasing depth. The HR-DGT fluxes of Fe measured in these profiles are closely related to the duration of anoxia at the corresponding points in the sediment (Supporting Information S.vii; Figs S2 and S3). This supports our earlier assessment that nitrate reduction makes a relatively small contribution to the overall organic matter mineralisation in this sediment. It also suggests that reductive dissolution is driving the release of Fe over time. Conversely, HR-DGT fluxes of Cu and Zn are highest at the SWI and decrease with depth. Such near surface mobilization has been linked to mineralisation of homogeneously distributed organic matter on the SWI^19, 20^. Although the unprecedented Pb fluxes are very low, they vary systematically and show a unique double maximum below the sediment water interface, meriting further study.

Caution should be exercised when comparing these trace metal profiles to those obtained for marine sediments using traditional techniques, which provide measurements of porewaters extracted from relatively large volumes of sediment (10–100 mL)^12, 13^. Our measurements at 0.28 mm intervals extend in total to only 16 mm into the sediment whereas the work of Klinkhammer, *et al*.^12^ provided data at 2–3 cm intervals, while the high resolution measurements of Shaw, *et al*.^13^ were at best 2.5 mm intervals. In our system, redox gradients, as exemplified by the Fe, Mn and O_2_ measurements, are tighter than those observed for the less reactive sediments of previous work. They do confirm more precisely, however, the previous suggestions of mobilisation of Cu at the sediment water interface and extend the observation to Zn. There is also visual evidence of some association between Ni and Co and Fe and Mn, which was found in some previous extended-scale profiles^13^ and attributed to Co and Mn being released from iron and manganese oxides as they are reductively dissolved.

The two-dimensional Fe and Mn HR-DGT flux data presented in Fig. 2 shows that there are three distinct areas where their fluxes are elevated: one at the SWI (*I*_x_, the ‘surface microniche’) and two below the SWI (*S*_A_ and *S*_B_, forthwith referred to collectively as ‘subsurface microniches’) (Table 1; Figs 2 and S4). Numerical modelling has shown that such well-defined features are likely to have arisen from locally increased porewater concentrations; desorption from small sediment volumes with high concentrations of exchangeable sorbed metal would be expected to show less steep gradients across the HR-DGT^38^. The standard deviations of HR-DGT fluxes of each trace metal in the anoxic sediment were used to provide a systematic definition of niches of increased metal supply. Areas where the HR-DGT fluxes of metals were two standard deviations above their respective averages in the anoxic background, and consisted of five or more contiguous measurement points, were defined as being indicative of a “metal microniche”. We used the results from the redox-active metals, Fe and Mn, to designate three separate features with sufficient numbers of distinct measurements to give confidence that a localized process is supplying metal for uptake by the HR-DGT, rather than a single isolated measurement that may have been caused by an experimental or analytical artefact^9^. This approach limited the detection of microniches to those that supply solute to areas of HR-DGT greater than 0.7 mm^2^, but provided features with sufficient measurement points to enable comparisons of trace metal release rates. We did not observe any differences in trace metal release that could be associated with the brief oxygenation during the sensor mounting and immediately following the start of the deployment.

### Trace metal fluxes at the surface microniche

*I*_x_ covered an area of 7.41 mm^2^ on the HR-DGT and overlapped with the position of the *Spirulina* aggregate and the observed O_2_ depletion. The average Fe HR-DGT flux within it was ~11 times higher than the average background in the oxic sediment and higher than in the two subsurface microniches (*p* < 0.05, Table 1). Approximately 64% of the microniche area was observed below the SWI at the position of the *Spirulina* aggregate (Fig. 2; Fig. S4). Average Pb and Mn HR-DGT fluxes within *I*_x_ were also higher than their respective fluxes in the top 1 mm of the sediment (6.3× and 1.44×, respectively) and significantly higher than those from the subsurface microniches (Table 1). Given the small proportion of the aggregate mineralised during the deployment (<5%), we do not believe that metal in the aggregate made a significant contribution to the HR-DGT fluxes measured at *I*_x_.

Previous studies have reported high dissolved Fe concentrations below the SWI in oxic sediments subject to episodic POM deposition^35, 36^, while others have demonstrated how the deposition of organic material as a homogeneous treatment onto the SWI resulted in mobilization of Mn, Co and As^18^. In these studies, bioturbation, stabilisation of reduced Fe(II) by organic matter ligands and changing redox status in the sediment, associated with organic matter mineralisation were suggested as possible causes. Our results show that the localized O_2_ depletion associated with a single aggregate of organic matter appears to be driving the co-located release of Fe, Mn and Pb above background levels across the SWI, and provide an alternative, complementary, explanation to the observations in these studies. This explanation could also account for the heterogeneity in dissolved Fe and Mn concentrations reported by Homoky, *et al*.^35^. The total mass of Fe bound by the HR-DGT resin within the area of *I*_x_ above the SWI (2.67 mm^2^) was 548 pmol. We believe that this Fe originated mainly from the reductive dissolution of Fe (oxyhydr)oxide minerals in the underlying sediment, following the emergence of localized anoxia. Given the small scale of the microniche and its position adjacent to the DGT device, DGT will have captured most, but not all of the flux of Fe attributable to the microniche. If we make the highly conservative assumptions that (1) this mass of Fe is the total amount that was released from the sediment to the water overlying the SWI, and (2) the anoxic microniche only lasted for the observed 32 h that *I*_x_ was anoxic, then the average rate of Fe release from the sediment during that time would have been at least 17 pmol h^−1^. Homoky, *et al*.^37^ (and references therein) report a range of Fe fluxes from sediments between 4.22–416.67 nmol m^−2^ h^−1^. Using our estimated average Fe release rate induced by the *Spirulina* aggregate, the previously reported range of Fe fluxes could be matched by between 248–24,510 similar aggregates (0.042–5.86 mol C m^−2^, Supporting Information, S1.vi) deposited on one square metre of sediment. Over the 32 h, this corresponds to a range of particulate organic C (POC) fluxes between 1.32 and 183.06 mmol m^2^ h^−1^. While previous studies suggest that these POC deposition rates are most representative of either shallow coastal conditions^39^, or periods of peak production under Arctic sea ice^40^, lower rates (such as those observed over deep sea sediments^39^), may also make a significant contribution to Fe release rates elsewhere (*c.f*. Homoky, *et al*.^35^). The HR-DGT is likely to have sampled only a part of the total Fe released from the sediment and it is highly likely the localized anoxic conditions would have persisted longer had we not ended the experiment, therefore our calculated Fe release rate is probably an underestimate.

Lalonde, *et al*.^41^ showed that around 21.5% of the organic C in sediments is incorporated with reactive Fe minerals. In our experiment, the reductive dissolution of Fe solid phases at the SWI may liberate further reactive organic C^42^, thus creating a partially self-sustaining transient diagenetic hot spot. Using the previously quoted organic C:Fe ratio of 4 ^41^, the above rates of Fe mobilization can be used to estimate a corresponding range of dissolved organic C release rates of 0.017–1.67 nmol m^−2^ h^−1^, which could make a significant contribution to previously reported rates of DOC diffusion from marine sediments^43^. Moreover, given that the organic matter existing in mineral-organic associations in sediments is often enriched in nitrogen^41^ and the affinity that species of phosphorus and arsenic can have for Fe (oxyhydr)oxide minerals^44–46^, these types of anoxic micro-features may also make a, hitherto unappreciated, contribution to the cycling of these elements at the SWI.

When considering these estimates, the following caveats should be considered. The organic matter content of this sediment (4.65%wt, Table S1) is more representative of a coastal sediment and higher than many deep-sea marine sediments^47, 48^. Therefore, the local populations of microorganisms may have been adapted to rapidly mineralise the aggregate organic matter. The ambient temperature in this experiment was between 5–15 °C higher than in most benthic environments, resulting in faster rates of organic matter mineralisation, but this will be offset to some extent by decreased diffusion coefficients of dissolved TEAs (O_2_, NO_3_^−^, SO_4_^2−^) at the lower temperatures^6^, and often lower ambient dissolved O_2_ concentrations^37^: these would increase the likelihood of anoxic microniche formation. Moreover, we expect that spatial and temporal variations in the quantity (and quality) of the organic matter that is deposited on the sediment surface, as well as seafloor environments (*e.g*. different ambient O_2_ and/or NO_3_^−^ concentrations) would significantly affect the sizes and durations of anoxic microniches and the extent to which various TEAs are reduced therein. Further experiments, aiming to capture the complete cycles of transient anoxia, are clearly required to quantify accurately the cycling of Fe (and other elements) within microniches in different sediments. We believe processes of elemental mobilization observed and implied here are likely to make the most significant difference in relatively shallow oxic sediments where organic matter turnover is dominated by microbial mineralisation at the SWI, and in environments that experience (possibly seasonal) periods of high organic matter deposition^40, 36^.

A comparison of the relative fluxes of Fe and Mn from *I*_x_ shows that their rates of mobilization are closely related across the microniche (Table 2). The size of the Mn microniche at *I*_x_ (1.12 mm^2^) is somewhat smaller than that of Fe, presumably due to the lower Mn content of the sediment compared to Fe (Table S1). The correlation between Fe and Pb fluxes is also statistically significant (*p* < 0.01)(Table 2), an association that is well established^49^. It is therefore likely that reductive dissolution of Fe (oxyhydr)oxide minerals is driving the Pb release across *I*_x_. Stahl, *et al*.^28^ found a peak Pb release of ~1.5 fmol cm^−2^ s^−1^ (~1.6× higher than at *I*_x_) during a 6 h deployment at the location of an artificial worm burrow which was closely associated with increased O_2_ concentrations inside it. They suggested that Pb was released by local oxidation of Pb sulphide minerals. This process is unlikely to have determined Pb mobilization here, because O_2_ was diminished at *I*_x_.

**Table 2:** Correlation (Pearson’s, *R* ^2^) between different metal fluxes in the surface microniche, *I*_x_, and the subsurface microniches, *S*_A_ and *S*_B_.

Surface Microniche, *I*_X_							
	Fe	Co	Ni	Cu	Zn	Pb	
Mn	**0.866**	0.124	0.100	0.004	0.058	0.287	*I*_X_ (*n* = 53; *p* < 0.01 for *R* ^2^ > 0.354)
Fe	×	0.322	0.248	0.008	0.006	**0.490 (0.839)**	
Co	**−**	×	**0.887**	0.017	0.164	0.297	
Ni	**−**	**−**	×	0.002	0.122	0.173	
Cu	**−**	**−**	**−**	×	0.000	0.067	
Zn	**−**	**−**	**−**	**−**	×	0.102	
**Subsurface Microniches**, ***S***_**A**_ **and** ***S***_**B**_							
	**Mn**	**Fe**	**Co**	**Ni**	**Zn**	**Pb**	
Mn	×	**0.958**	**0.962**	0.512	0.537	0.140	*S*_A_ (*n* = 11; *p* < 0.01 for *R* ^2^ > 0.735)
Fe	**0.962**	×	**0.951**	0.484	0.568	0.096	
Co	**0.986**	**0.955**	×	0.632	0.531	0.191	
Ni	**0.961**	**0.933**	**0.981**	×	0.065	0.712	
Zn	**0.761**	**0.825**	**0.774**	**0.811**	×	0.033	
Pb	**0.593**	**0.588**	**0.604**	**0.672**	**0.704**	×	
	*S*_B_ (*n* = 18; *p* < 0.01 for *R* ^2^ > 0.530)						

Bold print indicates where there is significant relationship between the fluxes (*p* < 0.01). Data points used to compare metal release in *I*_*X*_, *S*_*A*_ and *S*_*B*_ were selected using areas of high Fe mobilization. The value in brackets for Fe vs Pb indicates the correlation where six points are excluded from the comparison. Cu fluxes were not considered in *S*_A_ and *S*_B_ because they were below the method detection limit.

**Figure 3:**
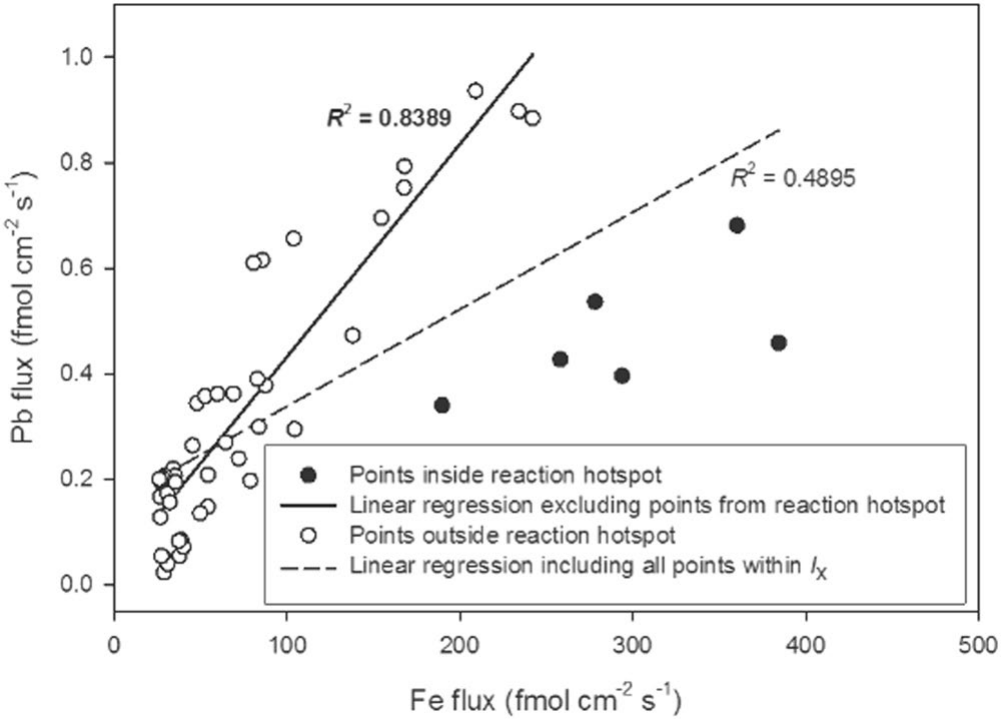
Time-averaged Fe flux vs. time-averaged Pb flux at individual analysis points inside the designated area of the surface microniche, *I*_x_. The two linear regressions, and corresponding *R* ^2^ values, show the correlation between Fe and Pb fluxes with and without the points inside the reaction hotspot (dashed and solid black lines, respectively).

The relationship between Fe and Pb, while statistically significant, is not consistent across *I*_x_. When the fluxes of the two metals are compared against each other, there appear to be two antagonistic mechanisms operating: one mobilizes both Fe and Pb at closely related rates, while another limits Pb flux into the HR-DGT when Fe fluxes are high (Fig. 3). This relative limitation can be identified by a feature defined by six contiguous measurement points at the centre of the *I*_x_ microniche: a possible ‘reaction hotspot’ (Figs 2 and 3). Rapid organic matter mineralisation has been linked to the formation of sulphide, S(-II), ‘hotspots’ in otherwise sulphide-free sediments^50, 51^. Given that Pb is thought to react with S(-II) much faster than Fe or Mn^52^, the likely explanation for the reduced Pb flux in the heart of the microniche is that, at some point during the deployment, the concentrations of Pb^2+^ and S(-II) increased past a level where the rate of PbS precipitation exceeded the rate of Pb^2+^ release by Fe mineral dissolution.

When compared to their average fluxes in the top 1 mm of the sediment, the fluxes of Co, Ni and Zn within the area of *I*_x_ (as defined by Fe measurements) were lower than in background oxic sediment, indicating that there was a process removing these metals from solution (Table 1; Fig. 2). The lower flux of these metals to the HR-DGT resin may be caused by different processes working independently or together. A localized decrease in H^+^ ion activity caused by their consumption in the dissimilatory reduction of Fe minerals^53^ could promote immobilization of these metals; however, such an effect has not been demonstrated at this spatial resolution and under these conditions. Zn is also known to react rapidly with S(-II)^52^. Given the higher concentrations of Zn in the pore water, it is possible that a high rate of ZnS mineral formation introduced a localized sink for S(-II) within *I*_x_. While Co and Ni do not react rapidly with S(-II), they are thought to be easily incorporated into sulphide minerals^52^. However, their cycling has also been linked to those of Mn oxide minerals^54, 55^. The combination of a good correlation between Co or Ni fluxes, with no apparent relationship between either metal and Mn (Table 2), suggests that the former process is more likely. Binding by organic ligands^56^ or removal from solution by locally proliferating micro-organisms^57, 58^ are also possible mechanisms that could reduce these metals’ flux into the HR-DGT. It could be speculated that, if the formation of S(-II) is the dominant process governing the reduced fluxes of these metals, the extent to which S(-II) was formed is greater than indicated by the Pb results alone.

### Trace metal fluxes from subsurface microniches

The location of the two subsurface microniches, *S*_A_ and *S*_B_, are shown in Fig. 2. They were not part of the experimental design, but the presence of such features has been attributed to bioturbation^9^. Within them, the fluxes of all metals were higher than the background, with the exception of Cu. Microniche *S*_A_ can be defined by above background fluxes of Mn, Fe and Zn; microniche S_B_ by fluxes of Mn, Fe, Co, Ni and Zn (Table 1).

Microniches *S*_A_ and *S*_B_ are comparable in terms of size (Fe microniche sizes: 1.54 and 2.52 mm^2^, respectively) and amounts of Fe and Mn mobilized. The Fe microniches were defined by a slightly larger number of points than the corresponding microniches of the other metals, therefore these Fe points were used to analyse the correlations between all metal fluxes at these locations. The simultaneous mobilization of Mn and Co in marine sediments has been previously linked to redox cycling of the Mn minerals^19, 20, 32^. The relatively sharp features showing elevated Fe and Mn fluxes and strong relationships between their fluxes and those of Co, suggest that this is also the case in *S*_A_ and *S*_B_ (Table 2)^38^. However, the significant differences between average fluxes of Co, Ni, and Zn in *S*_A_ and *S*_B_ (Table 1) could indicate that these redox processes operated at different rates, thereby influencing the fluxes of these metals from what otherwise appear to be similar microniches. It is also possible that different amounts of these metals were present at *S*_A_ and *S*_B_. Previous studies have found that the reactivity of the organic matter in microniches can be an important parameter in determining conditions where and when S(-II) formation and the subsequent precipitation of metals as sulphide minerals occurs^6, 51^. The organic matter introduced with the *Spirulina* aggregate was relatively fresh and highly labile. If oxidation of organic matter in the subsurface microniches is driving the release of metals here, they are likely to have been present in the sediment for some time before the start of the deployment and therefore not as fresh and or labile as at *I*_x_. As the surrounding environment is anoxic, this less reactive organic matter can still achieve similar reducing conditions to *I*_x_. The good correlation between Fe and Mn, and the poor correlation between Fe and Ni, Zn and Pb in *S*_A_ are indicative of this, while in *S*_B_ the correlations are consistently strong between all the metals that could be measured. This could indicate that the organic matter in *S*_A_ is more reactive than in *S*_B_ and that precipitation of insoluble sulphide minerals reduced the fluxes of some metals in *S*_A_. However, there are other possible reasons for the differences observed between these two sub-surface microniches. Microniches that have a low porosity will have reduced rates of diffusion within them and therefore are more likely to contain more reducing conditions than their surrounding environment^6, 21^. The combination of different porosities and types of organic matter in these microniches is also possible. As noted for *I*_x_, the possibility that labile trace metals are rendered partially or wholly unavailable for DGT uptake by organic ligands or microorganisms may also apply here, albeit to a lesser extent than at *I*_x_.

### Organic matter deposition at the SWI and its implications for elemental cycling in sediments

We have shown for the first time that deposition of organic matter and the resulting highly localized anoxic conditions have brought about intense mobilization of Fe and Mn at the SWI, proving part of our initial hypothesis. However, rather than observing release of some metals (*e.g*. Zn, Ni and Co) as the oxides are reductively dissolved, there is a net removal, presumably due to localised formation of S(-II) within the microniche, as established elsewhere^51^. We expect that under faster rates of mineralisation and/or higher concentrations of sulphate, the pyritization of Pb would be more extensive and a greater removal of soluble Fe may be observed. The intense release of Fe that we measured from the anoxic microniche suggests that the cycles of other elements (and their isotopes), thought to be associated with redox-active Fe minerals (*e.g*. C, N, P and S, as well as trace elements such as: Co, Ni, Cu, Zn and As), may also be indirectly linked to these localized features to a greater extent than previously realized; however, their magnitude should be more accurately quantified through targeted experiments across a wider range of environments. Anoxic micro-features, such as the one seen at *I*_x_, have previously been observed both *in-situ* and in laboratory mesocosms^4, 6, 11, 29, 59^ and modelling has shown that localised decomposition arising from settling organic matter can effect increased rates of nutrient cycling^6^. Here we demonstrate that this may also be an important factor in processes of early diagenesis, where new mineral phases (*e.g*. PbS and ZnS, possibly incorporating Ni and Co) are created, and existing ones (*e.g*. Fe and Mn (oxyhydr)oxides) are lost or modified simultaneously in small volumes. The role of anoxic microniches should be duly considered in future studies seeking to developing elemental budgets at the SWI, such as those recommended by Homoky, *et al*.^37^

Our results also provide circumstantial evidence that the translocation of labile organic matter from the SWI by faunal activity can move microbial mineralisation and the associated biogeochemical cycling to deeper in the sediments. This process has been implicated previously when hotspots of trace metal and S(-II) release have been identified^19, 32, 50^. Widerlund and Davison^50^ measured over 3000 S(-II) microniches in eutrophic lake sediments sampled over a five-month period, where an increase in S(-II) production following organic matter deposition contributed as much as 8% of the total S(-II) fluxes in the sediment. The authors suggested that the increased fluxes arose due to increased microbial activity, associated with the greater lability and/or size of the organic matter, as well as bioturbation^50^, which has been corroborated by subsequent numerical modelling^6, 51^. The microniches observed in that work were closer to a spherical geometry than at *S*_A_ and *S*_B_, but for the most part of a similar size (1–2.5 mm^2^ on the DGT gel). This range also fits within size ranges of fecal aggregates found in burrows in marine sediments, at depths of up to 20 cm below the SWI^5, 19^. Others have observed high CO_2_ concentrations and low pH around both ventilated and unventilated burrows below the SWI, which were also thought to reflect locally increased microbial activity associated with reactive organic matter^60, 61^. Subsequent work showed sediment micro-features of Fe and other trace metals around similar sediment features^28, 62^. Although action by biota during the deployment clearly influenced some parts of the sediment in our experiment, we did not directly observe any evidence of burrows at the HR-DGT probe interface. Yet, the elongated nature of the two subsurface microniches here bears some visual resemblance to the previously observed burrow structures^28, 60, 61^, so we cannot rule out the possibility that a previously existing burrow (or burrows) within the area of analysis may have collapsed during the sediment collection and/or subsequent resettlement, thereby trapping fecal, or other reactive organic matter below the SWI. Numerical modelling of solute transport and reactions associated with burrows has suggested that these features can have significant effects on the fluxes of C and N from (and within) sediments, depending on factors, such as their depth and frequency, and the reactivity of the sediment^63^. Our results provide further evidence indicating that effects of bioturbation extend to organic matter mineralisation and trace metal dynamics both at, and below, the SWI. This processing may also affect the occurrence and/or duration of anoxic microniches at the SWI and, hence, the balance between microbial processing (and associated elemental cycling) between the oxic and the anoxic parts of the sediment. Understanding this balance should be a key objective for future studies that seek to develop further insight into elemental cycling in surface sediments.

## Material and Methods

### Sediment handling and characterisation

Due to the difficulties associated with sampling deep-sea sediments, we chose to use a coastal sediment for this study. The sediment and overlying seawater were collected from Workington Marina, (UK) (54°38′52.54″N; 3°33′57.52″W) in April 2009 and stored in two 50 L plastic containers at 15–17 °C. The O_2_ concentration in the overlying water was maintained at 100% air sat. At the time of sampling, sediment was inhabited by various macrofauna species (*e.g. Corophium sp*. and juvenile polychaetes). In March 2010, the sediment was transferred into smaller acrylic aquaria (H × L × W: 15 × 14 × 8 cm: similar to one that had been used previously in other studies of oxygen dynamics at the SWI^6^), using a custom-made sediment sampler. The procedure maintained a virtually intact sediment structure. The setup was left for 2–3 d before the experiment was initiated, during which the overlying water inside the aquaria was circulated using pumps to ensure a steady semi-laminar flow of 2–3 cm s^−1^ and a constant O_2_ concentration at air saturation in the overlying water during the pre-incubation. The temperature was kept constant at 19 °C (±1). Active removal of visible fauna in the time leading up to the experiment aimed to minimize macrofauna activity during the experiments. The dissolved and solid fractions of the sediment samples underwent an extensive elemental characterisation for C, N, major cations and anions and trace metals (analyses are described in Section S1. iii). These were in good agreement with previously measured values in a marine sediment^20^ (Table S1). Five background sediment microelectrode profiles of H_2_S were measured at a resolution of 200 µm, using a Clark type H_2_S sensor^64^ connected to picoammeter (PA-2000, Unisense.dk). The profiles were measured at the centre of the mesocosm to avoid interfering with the rest of the experiment: no H_2_S was detected.

### Preparation of *Spirulina* aggregates and their characterisation

To form standardized labile particulate organic material, freeze dried *Spirulina* powder (SigmaAldrich.com) was mixed with a 0.5% agar paste (wt./wt.) made with ultra-pure water (Milli-Q™, Millipore; resistivity 18.2 MΩ·cm), rolled into spherical aggregates and dried. After drying, the aggregates had a diameter of 2–3 mm and a weight of 5–7 mg. Carbon mineralisation rates were analysed in subsets of *Spirulina* material and aggregates (Supporting Information, S.i), these were within previously observed values^65^ (Supporting Information, S.vi). Their total metal content was also analysed (Supporting Information, S.i). The Fe:C ratio in the aggregates was found to be between 24–54 µmol Fe:1 mol C, which is within the range of values previously measured in particulate organic matter collected from ocean water columns (Table S1)^65–68^; other metal concentrations were also representative of natural aggregates^69^.

### Preparation of the O_2_ sensitive optode foil and optode imaging

The principles for planar optode imaging have previously been described in detail^70–72^. The preparation of the O_2_-sensitive optode used here, and the details pertaining to the imaging, are described in detail in the Supporting Information (S1.ii). Briefly, The O_2_ sensitive sensing layer was coated directly onto a fibre optic faceplate measuring 75 × 50 × 3 mm (Schott.com)^73, 74^ and mounted into the removable sidewall of an acrylic aquarium (see later). The excitation light was delivered by a 10 W blue LED (light emitting diode; LZ4–40B200, LEDENGIN.com), with a peak wavelength of 465 nm. A CCD camera (Charged Couple Device) (SensiCam, PCO.de), mounted with a prime macro lens (Sigma 50 mm F2.8 EX DG Macro) was used to capture the emitted light. The macro lens and LED were equipped with the appropriate filters for dissolved oxygen measurements. The camera and LED operation was synchronized with a custom made trigger box controlled by the software Look@Molli^72^. The method detection limit (MDL) of the planar optode was determined at the end of the deployment to be 3.0 µM (see Supporting Information, S1.iv); herein this concentration is used as an operationally defined threshold for ‘*anoxic*’.

### Ultra-thin DGT gel preparation

Ultra-thin (50 µm-thick) resin gels sheets and calibration standards for the LA-ICP-MS were prepared following the method described in Lehto, *et al*.^31^. The details of this procedure are provided in the Supporting Information (S,iv). Hydrated resin gel sheet was trimmed to size (approx. dimensions: 50 × 70 mm) and placed between two sheets of acid washed Nuclepore membrane (pore size: 0.4 µm; thickness: 10 µm) (Whatman, Maidstone, U.K.). Herein, these three layers, specially designed for high-resolution measurements, are referred to as “High-Resolution DGT” (HR-DGT).

### HR-DGT-Planar Optode (HR-DGT-PO) sensor assembly and deployment

**Figure 4:**
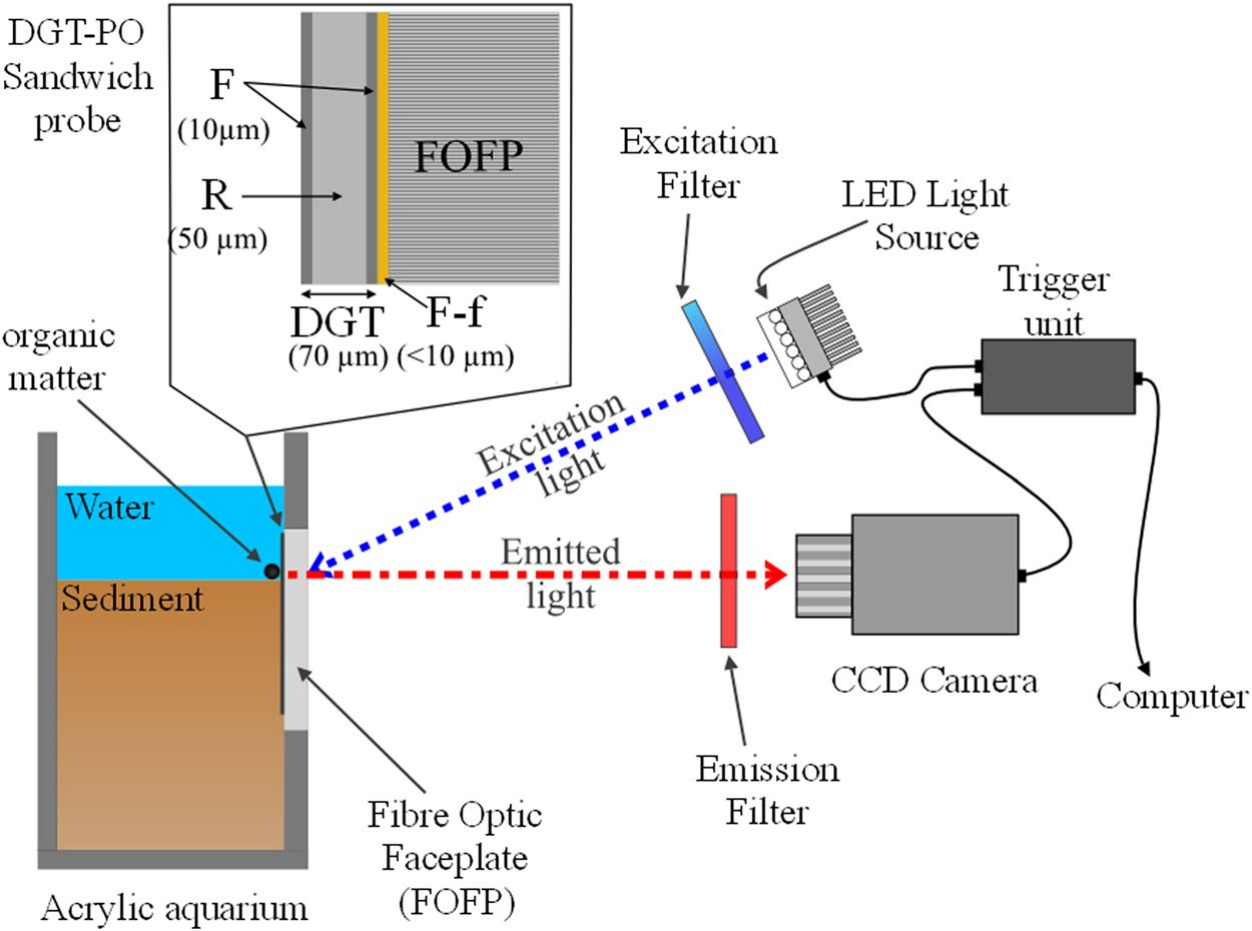
Experimental Set up (not to scale). Inset shows the DGT-Planar Optode (DGT-PO) sandwich probe mounted onto the Fiber Optic Faceplate (FOFP), including filter membrane (F), SPR-IDA resin (R) and PtTFPP- C545T sensing layer (F-p); thicknesses of the various components are shown in brackets.

The acrylic aquaria used in this work were designed with a replaceable sidewall (Fig. 4) allowing easy deployment of the HR-DGT-PO sensor, while ensuring minimal disturbance to the sediment structure. Prior to deployment, the sidewall containing the fibre optic faceplate was quickly wiped with 96% ethanol and rinsed with ultra-pure water. Using a pair of acid-washed plastic forceps, a 45 × 46 mm HR-DGT gel was laid flat on top of the fibre optic faceplate, facing the optode sensor. The HR-DGT was gently attached using water resistant vinyl tape (super 33+, Scotch®). Approximately 5 mm of the gel was covered by the vinyl tape on each side, leaving an exposed internal area of 40 × 42 mm.

Prior to deploying the HR-DGT-PO sensor, the water in the aquarium was drained, after which the aquarium was tilted back 15–20° to maintain the overall sediment structure and the sidewall was replaced with the sidewall containing the HR-DGT-PO sensor, ensuring a good contact between the sediment and the HR-DGT. The process of switching the sidewall took approximately 30 s, with a view to minimizing any oxygen contamination of the sediment. The aquarium was immediately filled with water, ensuring minimal disturbance of the sediment.

Immediately after deploying the sensor, two *Spirulina* aggregates and one aggregate of commercially available fish food of a similar size were gently placed in a row, at least 10 mm apart, on the water-sediment-HR-DGT interface using clean plastic forceps. The deployment was carried out for 44 h, during which the O_2_ concentration in the overlying water was monitored and the ambient temperature were kept at 19 °C (±1). The overlying water inside the aquaria was circulated and carefully flushed with air. Images of the mounted combined sensor were obtained at the start and at the end of the deployment, using ambient light to confirm the positions of the SWI and the aggregates on the optode images. Subsequent optode measurement images were recorded in darkness to avoid interference of ambient light at 20 min intervals.

At the end of the deployments, the combined sensor was retrieved by removing the sidewall as described above and the sensor was immediately rinsed thoroughly with MQ water to remove sediment particles. The HR-DGT was then cut out using a PTFE-coated razorblade and placed between two acid-washed plastic sheets for subsequent storage at 5 °C. The HR-DGT resin gels were dried within one week of the end of the deployment (see below).

### LA-ICP-MS analysis of the HR-DGT

Prior to analysis, the ultra-thin resin gel of the HR-DGT was separated from the Nuclepore membranes and dried using the method described by Lehto, *et al*.^31^. The gel was then trimmed (42 × 24 mm) targeting the SWI to fit into the ablation cell of the laser ablation unit and mounted onto a microscope slide using double-sided tape. An area of 40 × 23.22 mm was then analysed using laser ablation inductively coupled mass spectrometer (LA-ICP-MS) following the method used by Lehto, *et al*.^31^. The resolution of the measurements was 280 µm along the raster line, which was 100 µm wide. The interval between the centres of adjacent lines was 500 µm. C^13^, Rh^103^ and In^115^ were used as internal standards. Further details regarding the laser ablation of the gels can be found in the Supporting Information (S.v)

### Data analysis

The location of the ablation area with respect to the planar optode image was determined by measuring the exact distances from the edges of the gel. HR-DGT and O_2_ optode results are primarily focussed on a specific internal area of 15.1 × 23.22 mm (Fig. S1). The original spatial resolution of each discrete measurement point (‘pixel’) is 500 × 280 µm (HR-DGT) and 37 × 37 µm (planar optode); however, the resolution of the planar optode images was decreased to match that of the HR-DGT to allow for direct comparison between the two. All calibrated images were saved in TIFF file formats and were further processed using ImageJ. The presented trace metal images are artificially increased in size from the original 41 × 84 to 225 × 348 for visualisation purposes, while avoiding interpolation between data points; all data treatment is based on the original pixel size.

Statistical analysis of the metal fluxes from the microniches were carried out using MS Excel 2016 (correlations) and IBM SPSS Statistics for Windows (Version 22.0. Armonk, NY: IBM Corp) (one-way analysis of variance, ANOVA, with Tukey’s post-hoc test to compare means); the level of significance was 0.05, unless stated otherwise.

### Data Availability Statement

The datasets generated during and/or analysed during the current study are available from the corresponding author on reasonable request.

## Electronic supplementary material

Supplementary Information
