# Supplementary material for: A mesocosm study of oxygen and trace metal dynamics in sediment microniches of reactive organic material
Source: Sci Rep. 2017 Sep 12;7:11369. doi: 10.1038/s41598-017-10179-3 (PMC5595988; doi:10.1038/s41598-017-10179-3)
Supplement: Supplementary file 1 — Supplementary Information [file 41598_2017_10179_MOESM1_ESM.pdf]

Supporting information for:

A mesocosm study of oxygen and trace metal dynamics in sediment microniches of reactive organic material.

Niklas J. Lehto[1,*], Morten Larsen [2,3], Hao Zhang[5], Ronnie N. Glud [2,3,4] and William Davison[5]

[1] Department of Soil and Physical Sciences, Lincoln University, Lincoln 7647, Christchurch, New Zealand

[2] Department of Biology, Nordic Centre for Earth Evolution (NordCEE), University of Southern Denmark, Odense M, Denmark

[3] Scottish Marine Institute, Scottish Association for Marine Science, Oban, Scotland,

[4] Department of Ocean and Environmental Sciences, Tokyo University of Marine Science and Technology, 4-5-7 Konan, Minato-ku, Tokyo 108-8477, Japan

[5] Lancaster Environment Centre, Lancaster University, Lancaster, United Kingdom

*Corresponding author: N.J. Lehto, Tel: +64 3 423 0796, Fax: +64 3 325 3607, Email: Niklas.Lehto@lincoln.ac.nz

S1. Supporting Information: Methods

S.i. *Spirulina and Sediment analyses*

Spirulina. Approximately 0.25 g of the air-dried *Spirulina*-agar mix was measured into five pre-cleaned, acid washed and pre-weighed 50 mL polypropylene digest tubes. Next, 2 mL of concentrated nitric acid (Aristar®) was added to each and the mixtures were left to imbibe overnight. The samples were digested in a microwave sample preparation system (CEM Mars 5, CEM Corp., Matthews, NC) as follows: the temperature was raised, first to 55 °C and held there for 10 min; then to 75 °C at which it was maintained for 10 min; and, finally, to 95 °C where it was held for 30 min. The samples were cooled to room temperature, after which ultrapure Milli-Q (Millipore) water (resistivity: 18.2 MΩ) was added to increase the sample mass to 50.000 g. The diluted digests were then filtered through pre-cleaned Puradisc Aqua 30 syringe filters (Pore size: 0.45 µm) for ICP-MS analysis.

The volume-specific O_2 consumption rate of the *Spirulina* material was measured in 3 mL glass vials (Exetainers, Labco.co.uk) each with 15 mg freeze-dried *Spirulina* material topped up with 0.2 µm filtered seawater. The O_2 concentrations were recorded by a 4 channel fiber optic O_2 meter (Firesting, pyroscience.com), via O_2 sensitive sensor spots placed inside each vial.

Sediment porewater. Porewater was extracted from three separate sediment samples by centrifuging the sediment at 3000 rpm for 15 min and filtered through pre-cleaned Puradisc Aqua 30 syringe filters (Pore size: 0.45 µm) (Whatman Ltd.) in an anaerobic cabinet, under a nitrogen atmosphere. A subsample was refrigerated and sent for DOC and major anion analysis at a contract laboratory (Centre for Ecology and Hydrology, Lancaster, U.K.). DOC was analyzed using a Formac$^{HT/TN}$ TOC/TN analyzer (Skalar Ltd., Wheldrake, U.K.) after being acidified with 3M HCl and sparged with oxygen. The anions were measured using a Dionex ICS2000 (Thermo Fisher, Sunnyvale, CA)

ion chromatograph. Another filtered subsample was acidified with nitric acid (Aristar® BDH) to achieve a final concentration of 0.1 M HNO_3 for trace metal analysis using ICP-MS (see below) and used for major cation and Fe analysis using ICP-OES (iCAP 6300, Thermo Fisher, Hemel Hempstead, U.K.)

Sediment nitric acid extraction. Sediment samples were air dried and sieved through a 2 mm sieve, prior to microwave-assisted extraction with concentrated nitric acid (Aristar® BDH) according to EPA Method 3051. Briefly, 0.5 g of sediment was left to imbibe overnight in 10 mL of HNO_3. A temperature-assisted extraction was carried out in a microwave sample preparation system (CEM Mars 5, CEM Corp., Matthews, NC). The micro-wave cycle consisted of increasing the sample temperature to 175 °C in 5.5 min where it was maintained for 4.5 min. At the end of the micro-wave cycle the samples were left to cool, after which the extracts were filtered through pre-cleaned Puradisc Aqua 30 syringe filters (Pore size: 0.45 µm) (Whatman Ltd.) and diluted to 50 mL with ultrapure Milli-Q (Millipore) water for subsequent ICP-MS analysis. A subsample of the air-dried sediment was analyzed for total organic carbon and nitrogen using a LECO TruSpec® TOC/TN analyzer (LECO Corp., St Joseph, MI).

Solution ICP-MS analysis. Eluents, solutions and digests were analyzed using an inductively coupled plasma mass spectrometer (ICP-MS) (Thermo Electron X-Series, Hemel Hempstead, UK). Sc^{45} , Rh^{103} and In^{115} were used as internal standards. The accuracy of the ICP-MS calibration was confirmed by using a certified reference material (CRM) SLRS-5 (Riverine Water, National Research Council, Ottawa, ON, Canada). Metal concentrations in the CRM measured during various points of sample run consistently had values within one standard deviation of the certified values.

S.ii. ***Preparation of the O_2 sensitive optode***

The O_2 sensitive optode relies on the commonly used O_2 quenchable luminophore Pt(II)-meso-Tetra(PentaFluorophenyl)Porphine (PtTFPP) (PtT975, frontier scientific, frontiersci.com) [1,2]. The brightness of the PtTFPP is significantly increased by using an antenna dye; this principle is referred to as light harvesting [3,4]. The antenna dye used in this study is the Coumarin C545T (557595, Sigmaaldrich.com). The antenna dye effectively collects the excitation light from the LED source and transfers the energy to the PtTFPP O_2 indicator. Furthermore, sensors based on the light harvesting principle can effectively be used in combination with 2-dimensional color ratiometric imaging techniques[4,5]. The sensor cocktail consisted of a mix of Coumarin C545T (2% wt/wt) and PtTFPP (1% wt/wt) in a 4% (wt/wt) polystyrene matrix (goodfellow.com) in toluene Mayr, et al. [3]. The sensing chemistry was coated directly onto a fiber optic faceplate (FOFP) measuring 75×50×3 mm (Schott.com) as described by Fischer and Wenzhöfer [2]. After evaporation the sensing layer had a thickness of ~10 µm. The faceplate was later inserted into the removable wall of the acrylic aquarium (see later). The use of the FOFP significantly increases the maximal spatial resolution that can be achieved with planar optode (PO) measurements[2] by eliminating light guidance inside the aquarium wall and sensor support foil. The PO was later covered by the HR-DGT binding gel forming a combined HR-DGT-PO sensor (see below).

S.iii. ***Planar optode imaging.***

The imaging system used in this study is similar to that used in previous studies[6-10] and is based on a fast gateable, 12-bit, peltier cooled CCD camera (Charged Couple Device) (SensiCam, PCO.de). The camera was mounted with a prime macro lens (Sigma 50 mm F2.8 EX DG Macro), equipped with a 600 nm long-pass dichroic color filter (UQG optics, UQGoptics.com). The excitation light was delivered from a 10 W blue LED (light emitting diode) with a peak wavelength of 465 nm (LZ4-40B200, LEDENGIN.com), equipped with a 475 nm short-pass dichroic color filter (UQG

optics, UQGoptics.com). The camera and LED power supply were synchronized with custom made trigger box controlled by the software Look@Molli[11]. Recorded images were calibrated using the CalMolli software using the phosphorescent lifetime recorded in each pixel. The lifetime signal was calibrated using the modified Stern-Volmer equation (Eq. 1)[4,12]

$$\frac{\tau}{\tau_0} = \left[\alpha + (1-\alpha)\left(\frac{1}{1+K_{SV}\cdot C} \right) \right] \qquad (1)$$

Where τ_0 is the phosphorescent lifetime in the absence of O_2 and τ is the lifetime in the presences of any given O_2 level (C), K_{SV} the Stern-Volmer quenching constant and α the non-quenchable fraction of the phosphorescent light. τ was derived from two well defined time frames configured by the Look@Molli software. Subsequent calibrations of the O_2 concentration in each pixel were performed with 2-point calibration applying an experimentally determined α value of 0.14. All images were recorded in darkness to avoid interference of ambient light. Images were recorded using a 16 image average to increase the signal to noise ratio. Images were recorded at 20 min intervals.

The method detection limit (MDL) of the O_2 optode in this experimental set up was estimated as 3.0 µM, using the formula $MDL=\psi\sigma$, where ψ is the one-tailed t-value ($p < 0.01$, $n = 3564$; $\psi = 2.326$) and σ is the standard deviation of the O_2 measurements in the sediment 6 mm below the SWI (Fig. S1, $n = 2926$) at the end of the deployment.

S.iv. ***Preparation of the ultra-thin DGT***

The ultra-thin resin gels used suspended particulate reagent (SPR) with a polystyrene divinylbenzene substrate, which has been chemically derivitized with imidodiacetate (IDA) functionality (CETAC Technologies Inc., USA). The SPR-IDA resin (bead diameter: 0.2 µm) is selective towards Co, Ni, Cu, Cd and Pb and has been tested under a range of experimental

conditions[13]. The resin was incorporated into a hydrogel matrix using established procedures [14,15]. Briefly, 10 mL of acrylamide (40%) (VWR International) and 2.5 mL of DGT cross-linker (DGT Research Ltd.) were mixed together. Subsequently 1 mL of this mixture was added to 1 mL of a 10% solution of SPR-IDA, and then 14 µL of 10% ammonium persulfate (Fisher Scientific UK Ltd.) and 4 µL of N,N,N',N'-tetramethylethylenediamine (TEMED) (Sigma-Aldrich, Inc.) were added. The gel solution was then immediately pipetted on to a glass plate, with a 50 µm-thick plastic spacer on 3 sides. A second glass plate was then immediately placed on top of the spacer and fastened to the underlying glass plate using plastic clips. The glass plate assembly containing the resin gel solution was placed in an oven for 1 h at 45°C. The plastic clips were removed from the glass plate assembly, which was then immersed in 0.5 L MQ water (Millipore) and allowed to hydrate for approximately 30 min. The glass plates were then separated and the resin gel removed from the assembly and placed into 0.5 L of MQ water. The gel was left to hydrate for 24 h, during which the water was changed several times.

S.v. *Laser Ablation Inductively-Coupled Plasma Mass Spectrometry (LA-ICP-MS) analysis*

Standards. Two sets of five calibration standards were prepared, with four replicates for each standard: one set of standards for Mn, Co, Ni, Cu, Zn, Pb (mass range 0 – 200 ng cm^{-2}) and another set for Fe (mass range 0 – 1000 ng cm^{-2}) according to the method presented by Lehto, et al. [15]. The mass of metal bound on three replicate standards was determined by eluting the metal using 1 mL of 1 M HNO_3 and analysing the eluent using ICP-MS in solution mode as before. Metal-specific elution factors determined by Warnken, et al. [13] were used to estimate the masses of each metal bound to a unit area of gel.

Sample and standard preparation before analysis*[15]*. The DGT resin gels were mounted on plates of 0.04 cm-thick DGT diffusive gel that had equilibrated with a solution containing 1 ppm Rh^{103} and In^{115}. The diffusive gel-resin gel stack was then dried onto a piece of Supor-450 filter membrane (0.45 µm pore size, 0.14 mm thickness, Pall Corporation, USA) and mounted on microscope slides using double-sided tape.

All handling and processing of gels, before and after deployment was carried out in a class-100 laminar flow hood using clean laboratory techniques, including acid washing of all equipment.

LA-ICP-MS analysis was undertaken using a NewWave UP-213 laser ablation unit (ESI, Cambridge, U.K.) with a Nd:YAG laser (213 nm), coupled to a Thermo Electron X Series (Hemel Hempstead, UK) ICP-MS. The analysis was run in line scan mode using a beam diameter of 100 µm, scanning speed of 50 µm s^{-1} and repetition rate of 20 Hz. C^{13}, Rh^{103} and In^{115} were used as internal standards to monitor and correct for instrumental drift. The laser power was optimized for these settings with a view to achieving the optimum metal (M) counts per second (cps) to internal standard (IS) cps ratio (M/IS) while avoiding ablation of the backing layer. The optimum laser power was 47.5%, which was specific to the laser ablation unit used for that time. The fluence at the sample was 0.02 J cm^{-2} and was monitored throughout the standard and sample analysis. The acquisition time of the ICP-MS was 215 ms, which provided readings every 11 µm of line ablation. The instrumental noise was smoothed by taking an average of 26 data acquisitions, therefore the resolution along the line of ablation was 280 µm (relative standard deviation of M/IS at this interval was less than 10 % for all the standards described below). The interval between the centers of adjacent lines was 500 µm. These settings were used in the analysis of the standards and the samples.

The standards were analyzed using LA-ICP-MS prior to each sample run by ablating five 2000 µm-long lines in each standard gel to obtain the relationship between counts per second (cps) and mass of metal bound by the DGT resin (ng cm^{-2}). Two standards were analyzed at the end of each sample run to further confirm instrument stability. The calibration curves for all the metals were linear across the concentration range. The relationship between M/IS and mass of metal bound by the DGT resin was determined using a bivariate line fitting algorithm [16]. The correlation coefficients (R^2) for all metals were greater than 0.95. The M/IS readings for the blank could not be resolved from the baseline, therefore the method detection limit was estimated using the next lowest standard (mass of metal bound: 10 ng cm^{-2}). The method detection limits for the metals (expressed as three times the standard deviation of the M/IS measured at the lowest standard, divided by the slope of the calibration curve, n = 35), expressed as equivalent HR-DGT fluxes over a 44h deployment time, were: Fe 1.46, Mn 1.80, Co 0.04, Ni 0.18, Cu 0.19, Zn 1.52 and Pb 0.01 fmol cm^{-2} s^{-1}. The correlation coefficients of the calibration curve, instrument stability and method detection limits were all consistent with previously published values where similar analysis has been carried out [17-19].

S2. Supporting Information: Results

S.vi. ***Estimate of the particulate organic carbon (POC) content of the Spirulina pellet and extent of mineralisation.***

We did not measure the total organic matter content of the pellets, however, using previously determined total mineral content of dry *Spirulina* material, we estimate that this will have been around 90% on a dry weight basis[20], which amounts to approximately 170 – 239 µmol C per pellet (assuming a POC to POM ratio of 1:2.2) [21].

The *Spirulina* material had an average volume-specific O_2 consumption rate of 377 nmol cm^{-3} h^{-1} (S.D = 109, n = 3), which is within the range of previous published values for O_2 uptake of marine aggregates/pellets (280 – 450 nmol cm^{-3} h^{-1}) [22]. The equivalent carbon mineralization rate was 314 nmol cm^{-3} h^{-1} [23]. Using the mass of *Spirulina* material in the incubations, we estimate a carbon-specific respiration constant of 1.843×10^{-3} h^{-1} and that that approximately 3.5% of the initial POM in the aggregates was mineralized during the deployment.

S.vii. ***Relating O_2 dynamics to Fe mobilisation.***

Planar Optodes measure the O_2 concentration at the device interface, while a HR-DGT gel samples a volume of sediment away from the device interface to a distance of <1 to 3 mm, determined by a combination of sediment and DGT parameters[24]. Bearing this in mind, the amount of redox-sensitive metal, such as Fe, measured by the HR-DGT resin at a given location is likely to be determined by a combination of four factors: (1) the total amount of Fe within the volume of sediment sampled (2) the rates at which immobile ferric minerals and dissolved ferrous Fe undergo reactions that determine the proportions of their respective species (*e.g.* dissolution/precipitation, sorption/desorption) (3) the length of time during the deployment when these reactions are likely to occur at that location and (4) the time it takes for the mobile species to diffuse into the HR-DGT.

Consideration of these factors suggests that comparing the time-integrated Fe flux into the DGT to a nominal (or average) O_2 concentration at a given location is unlikely to be meaningful. However, there is a basis for comparing the Fe flux to the amount of time during which the metal is likely to exist in a mobile species at that location. We did this using the average O_2 (optode) and Fe flux measurements (DGT) from the three background profiles and obtained a good correlation between Fe flux to DGT from the sediment and the amount of time that the corresponding sediment is anoxic (Fig. S3 a). The scatter in the cluster of data points corresponding to the maximum time probably reflects the heterogeneity in the sediment in terms of the variability in the Fe concentrations in the sediment and the differences in reactivity of the various iron minerals within the sediment [25-27]. Although there is an overall similar relationship between Fe flux and duration of anoxia in and around I_x (area shown in Fig S1), there are distinct variations (Fig. S3b). For this highly transient feature there is an extremely high degree of variability in Fe fluxes arising from parts of the sediment that were anoxic between 5 and 30 h.

S3. Supplementary Tables and Figures

Table S1. *Spirulina*, porewater and bulk sediment concentrations.

Analyte	*Spirulina* (*n* = 3) Average (S.D.)	Sediment Porewater (*n* = 3) Average (S.D.) (mg L^{-1})	Bulk sediment (*n* = 3) Average (S.D.) wt./wt. %
DOC/TOC[1]	n/a	1.42 (0.13)	4.65 (0.30)
TN[2]	n/a	n/a	0.29 (0.02)
Na^{+}	n/a	66 (0.75)	n/a
K^{+},	n/a	2.90 (0.05)	n/a
Ca^{2+}	n/a	2.90 (0.04)	n/a
Mg^{2+}	n/a	8.80 (0.11)	n/a
Cl^{-}	n/a	799.00 (152.00)	n/a
NO_3^{-}	n/a	0.07 (5×10^{-4})	n/a
SO_4^{2-}	n/a	33.80 (6.20)	n/a
Analyte (trace metals)	(µg g^{-1})[@]	(µg L^{-1})	(µg g^{-1})[@]
Mn	3.19 (0.25)	6,924.27 (177.26)	1,111.94 (87.45)
Fe	73.80 (5.58)	1,128.90 (61.85)	43,497.66 (5128.46)
Co	0.01 (0.002)	4.08 (0.12)	9.28 (1.56)
Ni	0.62 (0.002)	11.53 (0.16)	23.73 (2.51)
Cu	< 0.02[§]	4.01 (0.09)	28.29 (5.09)
Zn	4.74 (0.82)	8.20 (0.80)	158.95 (15.88)
Pb	< 0.02[§]	2.17 (0.03)	22.41 (4.30)

[1] Dissolved organic carbon (porewater) and total organic carbon (bulk sediment)
[2] Total nitrogen (bulk sediment only)
[@] trace metal content expressed as mass per gram dry weight (DW)
[§] Below method detection limit
n/a - not analyzed

Figure S1. Visual image (not to scale) of the full DGT area at the end of the deployment, showing: the ablated area (delineated by white and grey dotted line); areas of gel used to define the 'oxic' and 'anoxic background' metal fluxes (pale rectangles); the area where metal and O_2 results are shown (yellow rectangle); and the location of the three profiles used to compare bulk sediment O_2 concentrations and Fe fluxes (yellow lines).

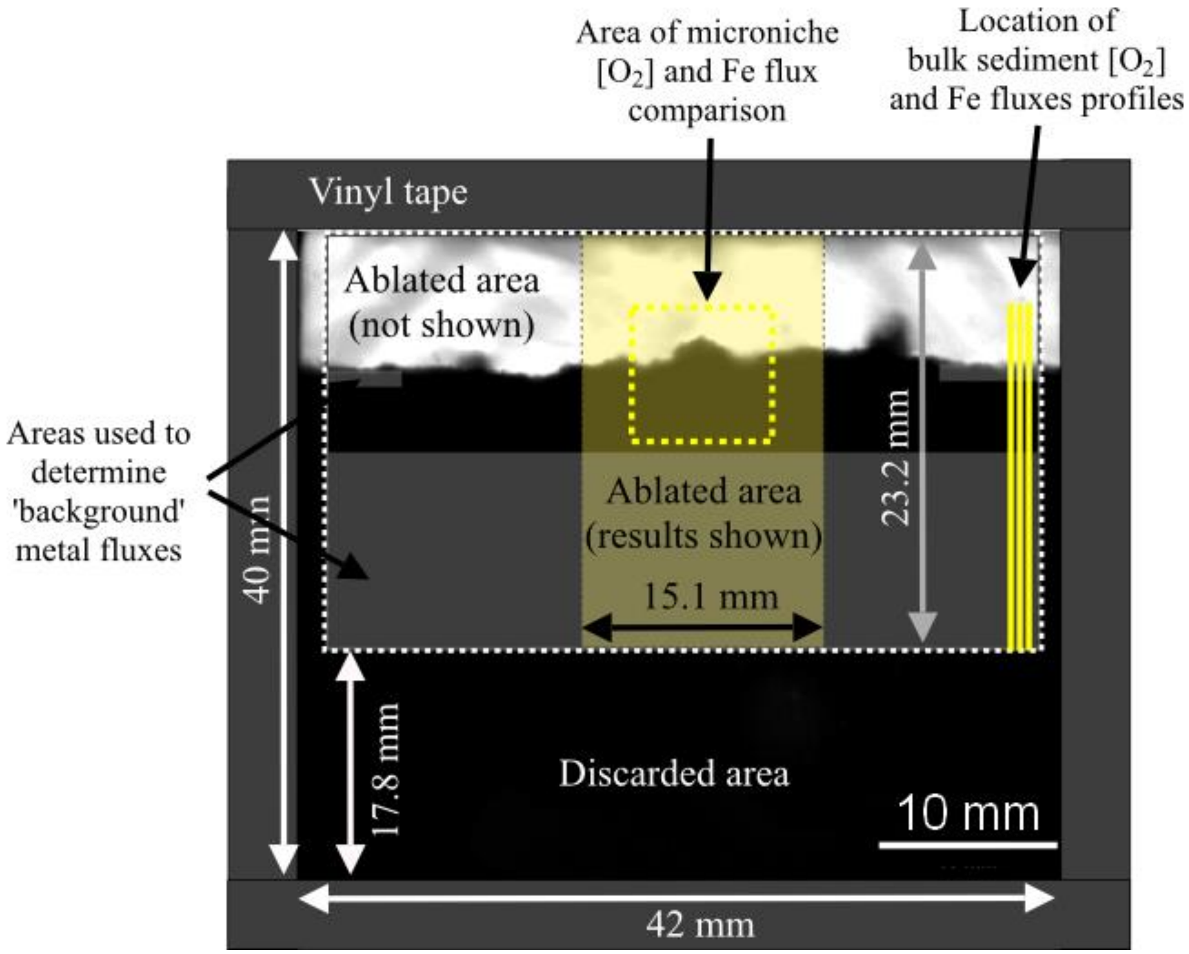

Figure S2. Average trace metal profiles in the background sediment (*n*=3). The *y*-axes show depth (mm), where 0 is the sediment-water interface. *x*-axes show metal flux (fmol cm^{-2} s^{-1}). Where shown, the vertical red dotted line indicates the method detection limit for the metal in question. “O2 Time” shows the average time where the average O_2 concentration is below 3.0 µmol L^{-1} (time in hours is shown on the *x*-axis); error bars show the standard deviation of three adjacent measurements).

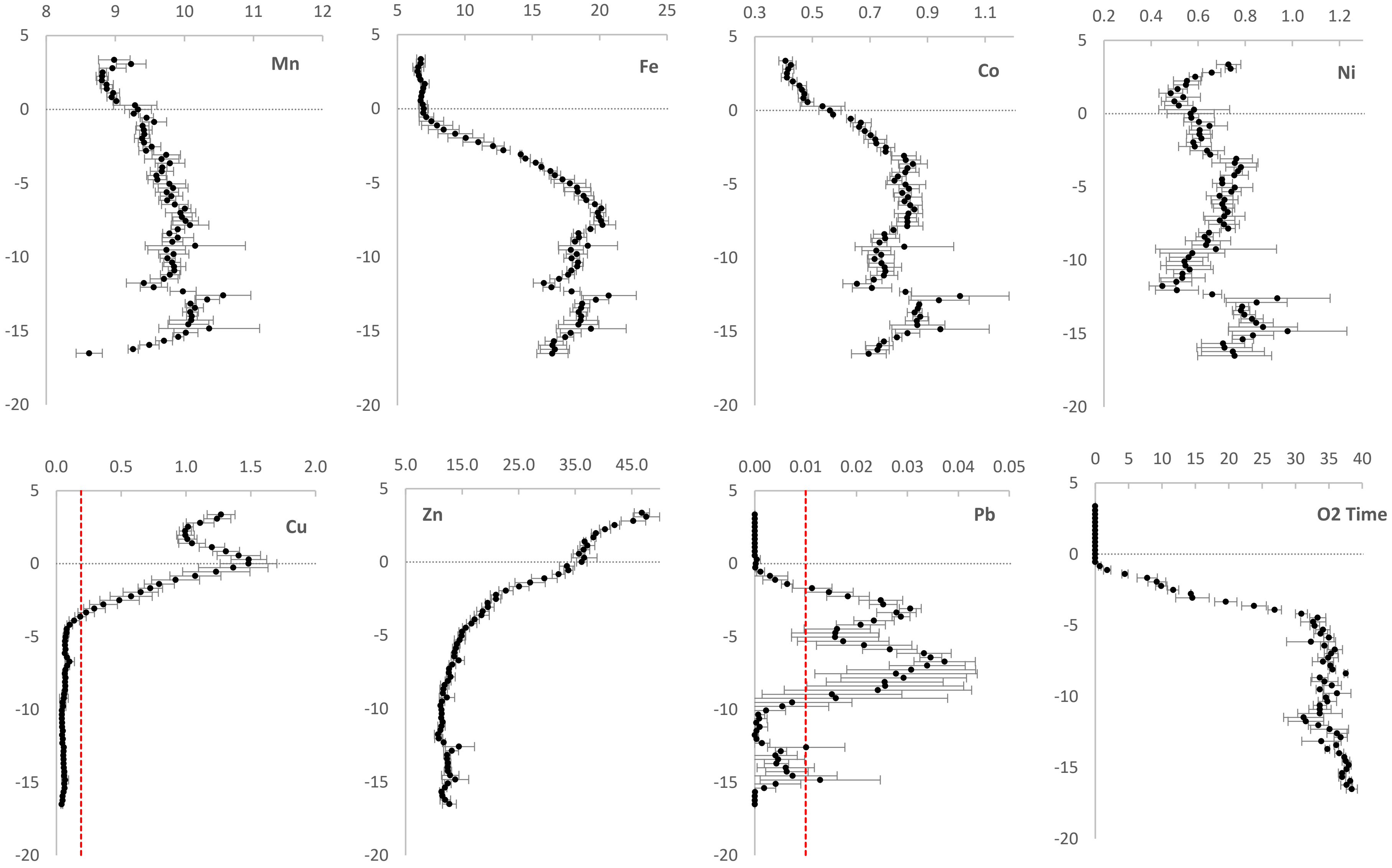

Figure S3. (a) Average Fe flux at three adjacent profiles at 280 µm depth intervals across the SWI vs. average time each interval was anoxic, determined from three adjacent profiles across the SWI; error bars show the standard deviation (n = 3). (b) Fe flux vs. duration of anoxia in the area around the surface microniche, I_x.

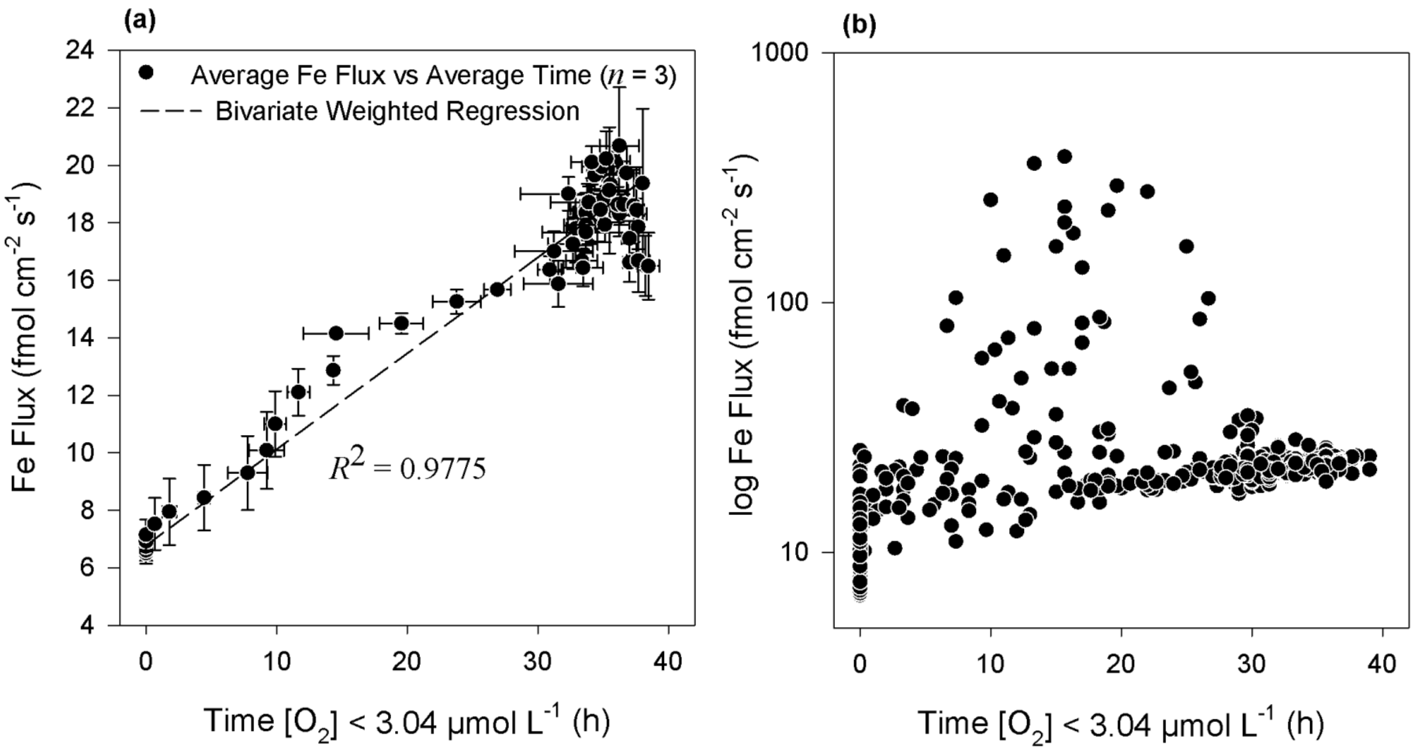

Figure S4. 3D representation of Fe fluxes across area of analysis

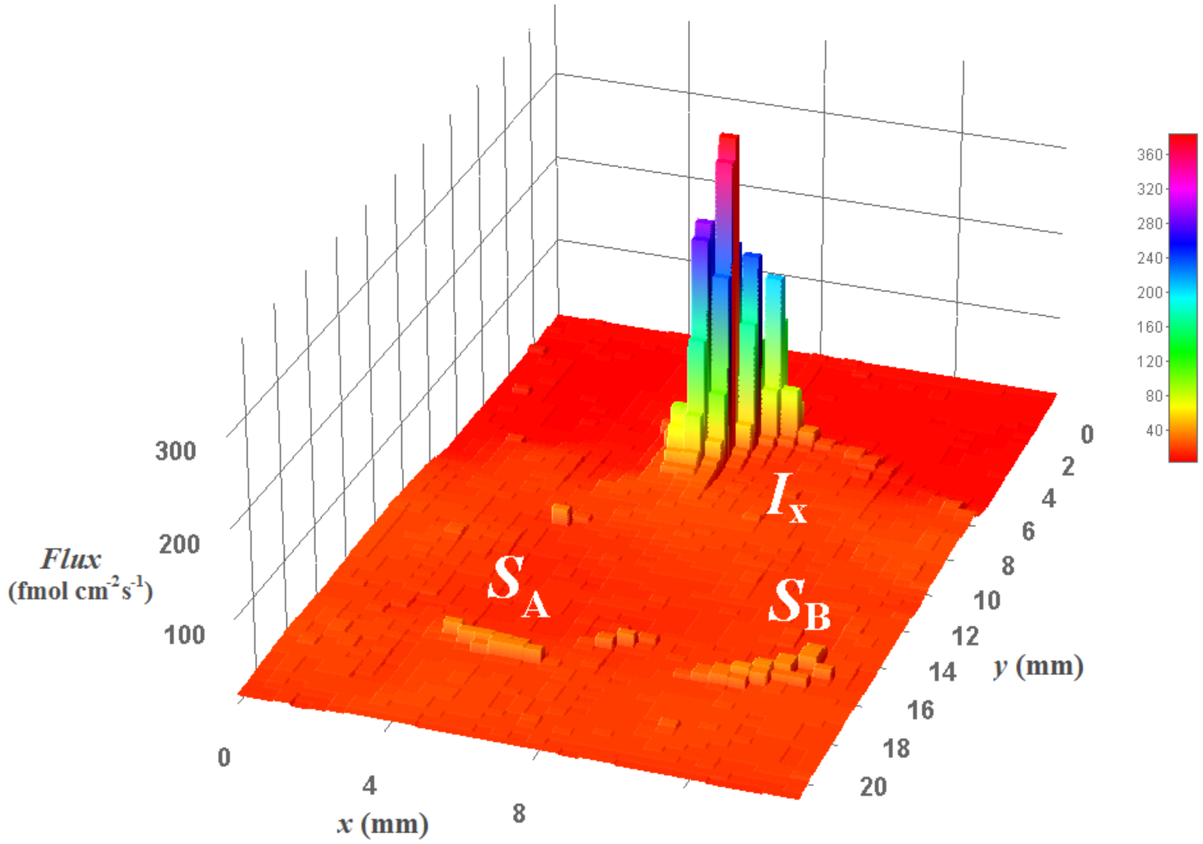

S4. References

1. Borisov, S. M. & Klimant, I., Luminescent nanobeads for optical sensing and imaging of dissolved oxygen. *Microchim. Acta* **164** (1), 7-15 (2008).
2. Fischer, J. P. & Wenzhöfer, F., A novel planar optode setup for concurrent oxygen and light field imaging: Application to a benthic phototrophic community. *Limnol. Oceanogr. Methods* **8** (6), 254-268 (2010).
3. Mayr, T. *et al.*, Light harvesting as a simple and versatile way to enhance brightness of luminescent sensors. *Anal. Chem.* **81** (15), 6541-6545 (2009).
4. Larsen, M., Borisov, S. M., Grunwald, B., Klimant, I., & Glud, R. N., A simple and inexpensive high resolution color ratiometric planar optode imaging approach: application to oxygen and pH sensing. *Limnol. Oceanogr. Methods* **9** (9), 348-360 (2011).
5. Santner, J., Larsen, M., Kreuzeder, A., & Glud, R. N., Two decades of chemical imaging of solutes in sediments and soils – a review. *Anal. Chim. Acta* **878**, 9-42 (2015).
6. Frederiksen, M. & Glud, R. N., Oxygen dynamics in the rhizosphere of Zostera marina: A two-dimensional planar optode study. *Limnol. Oceanogr.* **51**, 1072-1083 (2006).
7. Behrens, J. W., Stahl, H. J., Steffensen, J. F., & Glud, R. N., Oxygen dynamics around buried lesser sandeels Ammodytes tobianus (Linnaeus 1785): mode of ventilation and oxygen requirements. *J. Exp. Biol.* **210** (6), 1006-1014 (2007).
8. Volkenborn, N., Polerecky, L., Wethey, D., & Woodin, S., Oscillatory porewater bioadvection in marine sediments induced by hydraulic activities of Arenicola marina. *Limnol. Oceanogr.* **55** (3), 1231 (2010).
9. Stahl, H. *et al.*, A combined sensor for simultaneous high resolution 2-D imaging of oxygen and trace metals fluxes. *Limnol. Oceanogr. Methods* **10** (5), 389-401 (2012).
10. Lehto, N., Glud, R. N., Norði, G. á., Zhang, H., & Davison, W., Anoxic microniches in marine sediments induced by aggregate settlement: Biogeochemical dynamics and implications. *Biogeochemistry* **119** (1-3), 307-327 (2014).
11. Holst, G. & Grunwald, B., Luminescence lifetime imaging with transparent oxygen optodes. *Sens. Actuator B-Chem.* **74** (1), 78-90 (2001).
12. Klimant, I., Meyer, V., & Kühl, M., Fiber-optic oxygen microsensors, a new tool in aquatic biology. *Limnol. Oceanogr.* **40** (6), 1159-1165 (1995).
13. Warnken, K. W., Zhang, H., & Davison, W., Performance characteristics of suspended particulate reagent-iminodiacetate as a binding agent for diffusive gradients in thin films. *Anal. Chim. Acta* **508**, 41-51 (2004).
14. Davison, W., Fones, G., Harper, M., Teasdale, P., & Zhang, H. In situ environmental measurements using dialysis, DET and DGT (John Wiley & Sons, New York, NY, 2000), pp. 495-569.
15. Lehto, N. J., Davison, W., & Zhang, H., The use of ultra-thin diffusive gradients in thin-films (DGT) devices for the analysis of trace metal dynamics in soils and sediments: a measurement and modelling approach. *Environ. Chem.* **9** (4), 415-423 (2012).
16. York, D., Evensen, N. M., Martınez, M. L., & Delgado, J. D. B., Unified equations for the slope, intercept, and standard errors of the best straight line. *Am. J. Phys.* **72** (3), 367-375 (2004).

17. Warnken, K. W., Zhang, H., & Davison, W., Analysis of Polyacrylamide Gels for Trace Metals Using Diffusive Gradients in Thin Films and Laser Ablation Inductively Coupled Plasma Mass Spectrometry. *Anal. Chem.* **76**, 6077-6084 (2004).
18. Stockdale, A., Davison, W., & Zhang, H., Micro-scale biogeochemical heterogeneity in sediments: A review of available technology and observed evidence. *Earth-Sci. Rev.* **92**, 81–97 (2009).
19. Gao, Y. & Lehto, N., A simple laser ablation ICPMS method for the determination of trace metals in a resin gel. *Talanta* **92**, 78-83 (2012).
20. Tokuşoglu, Ö. & Üunal, M., Biomass nutrient profiles of three microalgae: Spirulina platensis, Chlorella vulgaris, and Isochrisis galbana. *J. Food Sci.* **68** (4), 1144-1148 (2003).
21. Klaas, C. & Archer, D. E., Association of sinking organic matter with various types of mineral ballast in the deep sea: Implications for the rain ratio. *Global Biogeochem. Cycles* **16** (4), 63-61-63-14 (2002).
22. Ploug, H., Small-scale oxygen fluxes and remineralization in sinking aggregates. *Limnol. Oceanogr.* **46** (7), 1624-1631 (2001).
23. Ploug, H., Grossart, H. P., Azam, F., & Jørgensen, B. B., Photosynthesis, respiration and carbon turnover in sinking marine snow from surface waters of Southern California Bight: implications for the carbon cycle in the ocean. *Mar. Ecol. Prog. Ser.* **179**, 1-11 (1999).
24. Sochaczewski, Ł., Davison, W., Zhang, H., & Tych, W., Understanding small-scale features in DGT measurements in sediments. *Environ. Chem.* **6**, 477–485 (2009).
25. Motelica-Heino, M., Naylor, C., Zhang, H., & Davison, W., Simultaneous release of metals and sulphide in lacustrine sediment. *Environ. Sci. Technol.* **37**, 4374-4381 (2003).
26. Fones, G. R., Davison, W., & Hamilton-Taylor, J., The fine-scale remobilization of metals in the surface sediment of the North-East Atlantic. *Cont. Shelf Res.* **24** (13), 1485-1504 (2004).
27. Robertson, D., Welsh, D. T., & Teasdale, P. R., Investigating biogenic heterogeneity in coastal sediments with two-dimensional measurements of iron (II) and sulfide. *Environ. Chem.* **6** (1), 60-69 (2009).
